# Supplementary figures and images for: Glycemic control in newly insulin-initiated patients with type 2 diabetes mellitus: A retrospective follow-up study at a university hospital in Ethiopia
Source: PLoS One. 2022 May 26;17(5):e0268639. doi: 10.1371/journal.pone.0268639 (PMC9135271; doi:10.1371/journal.pone.0268639)

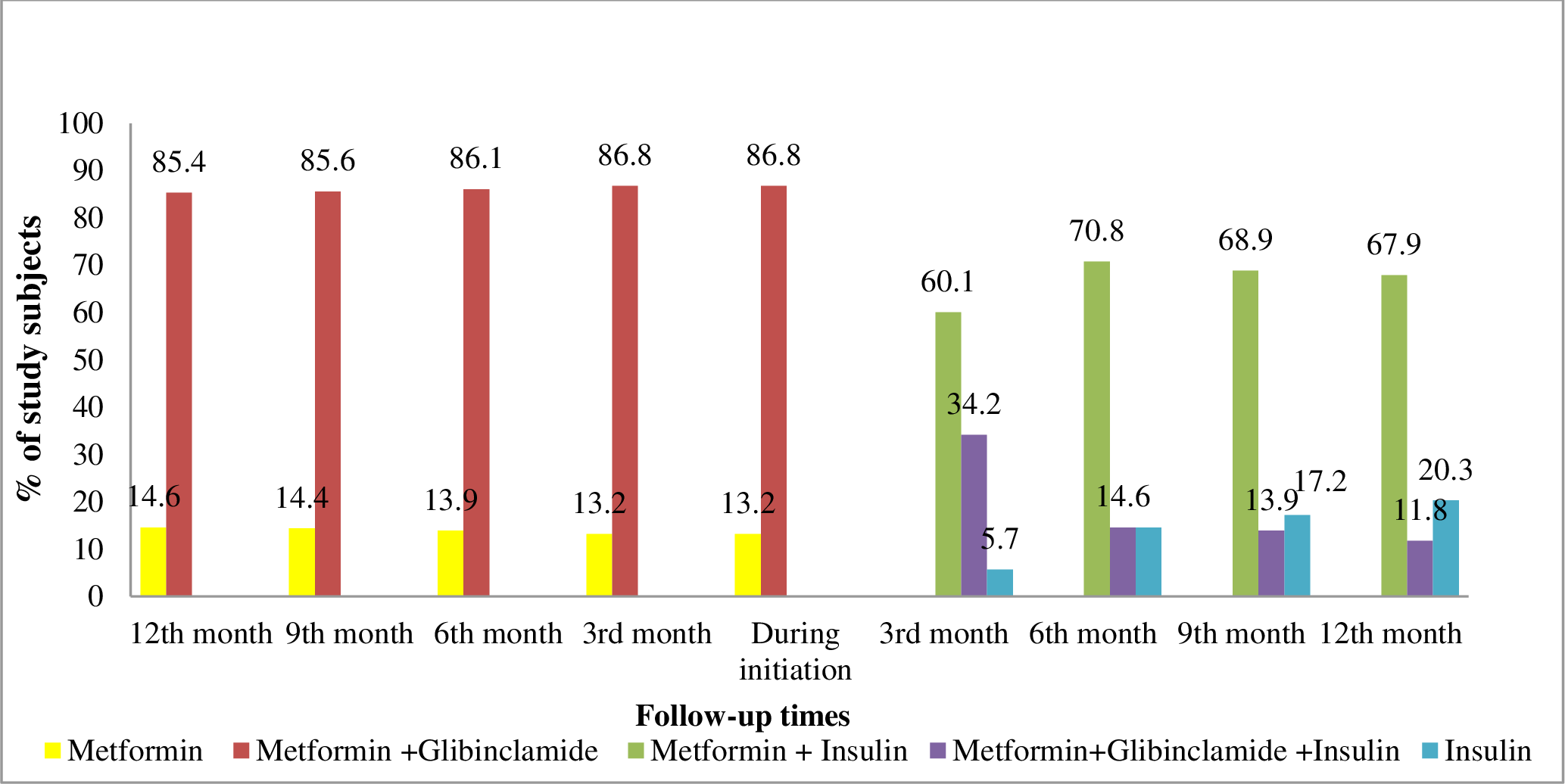

Supplement: S1 Fig — (TIF) [file pone.0268639.s001.tif]

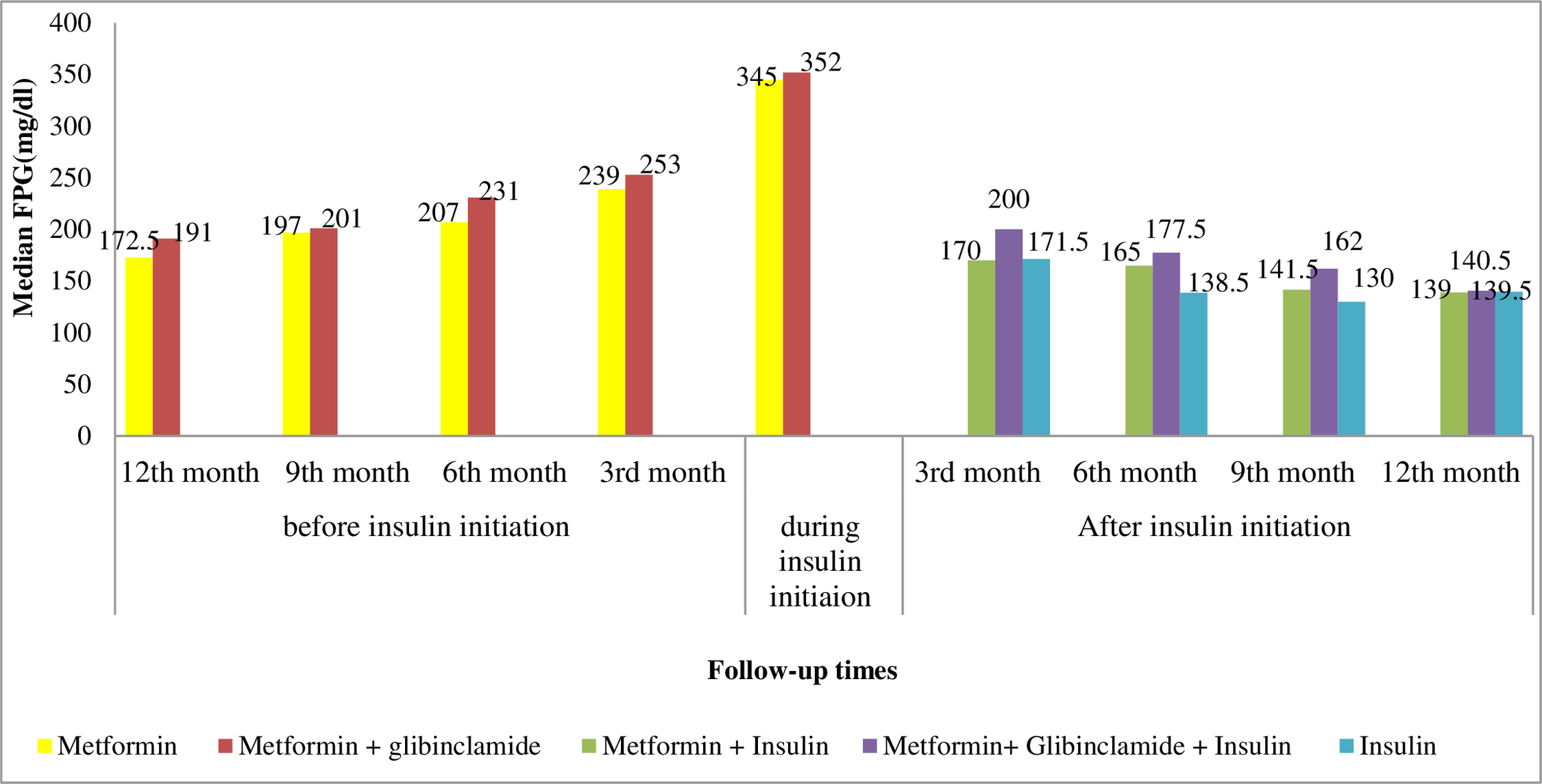

Supplement: S2 Fig — (TIF) [file pone.0268639.s002.tif]
